# ﻿*Astragalusliuaiminii*, a new species of *Astragalus* (Fabaceae) from Xinjiang, China

**DOI:** 10.3897/phytokeys.243.119707

**Published:** 2024-06-26

**Authors:** Zong-Zong Yang, Quan-Ru Liu, Zhen Liu, Ji-shan Xiang, Xiao-lan Li

**Affiliations:** 1 Yili Normal University/Xinjiang Key Laboratory of Lavender Conservation and Utilization, Xinjiang 830500, Yining, China Yili Normal University Yining China; 2 Xinjiang Ziranli Information Technology Co., Ltd, Xinjiang 830011, China Xinjiang Ziranli Information Technology Co., Ltd Xinjiang China; 3 College of Life Sciences, Beijing Normal University, Beijing 100875, China Beijing Normal University Beijing China

**Keywords:** *Astragalus* sect. *Laguropsis*, new species, taxonomy, Xinjiang

## Abstract

A new species, *Astragalusliuaiminii* Z. Z. Yang & Q. R. Liu (Fabaceae), is described and illustrated from Xinjiang Province, China. The new species is close to *A.wenquanensis* S. B. Ho, but differs from the latter by leaves having a single leaflet (vs. 3–5 leaflets), and inflorescences with 1–2 flowers (vs. inflorescences with 5–7 flowers). It is also similar to *A.monophyllus* Maxim in leaf shape, but differs by its calyx expanding to become saccate and totally enveloping the pod (vs. calyx tubular, and ruptured by pod after flowering).

## ﻿Introduction

The genus *Astragalus* Linnaeus is the largest genus of flowering plants, containing about 2500 to 3000 species, with ca. 2350 species in the Old World and ca. 500 species in the Americas (Chang et al. 2007). Species of the genus are mainly distributed in arid and semi-arid mountainous regions of the Northern Hemisphere, as well as in South America and Africa (Zhai and Yan 2010; Podlech and Zarre 2013). China is one of the largest centres of diversity for the genus with 388 species, of which 210 are endemic (Ho 1993; Chang et al. 2007; Xu and Podlech 2010). While conducting field work in June 2023, in the Habahe Region, we collected some interesting *Astragalus* specimens that have distinct characters including being acaulescent or nearly so, leaves with only one leaflet, inflorescence 1- to 2-flowered, and the calyx at first being tubular and enlarging after anthesis becoming saccate. After critical study of the specimens and comparison with other existing species in the surrounding area, we confirmed that these specimens were new to science. The new species belongs to Astragalussect.Laguropsis (Podlech and Zarre 2013), and is described and illustrated below.

## ﻿Materials and methods

Specimens were collected from Habahe County of Xinjiang Province. Morphological studies of the new species were based on observation of living individuals. Comparisons of the new species with other related specimens were conducted by checking materials from PE and XJBI, as well as virtual specimen databases (CCAU, KUN, IBK, IBSC, CVH and JSTOR). Measurements were carried out under a stereomicroscope (Olympus SZX2, Tokyo, Japan) using a ruler and a metric vernier caliper.

## ﻿Taxonomy

### ﻿ Astragalus liuaiminii

Taxon classificationPlantaeFabalesFabaceae

Z.Z.Yang & Q.R.Liu sp. nov.

3B46F85C-7D64-559F-8EF9-AD22D883C5A6

urn:lsid:ipni.org:names:77344331-1

Figs 1 , 2 , 3

#### Diagnosis.

*Astragalusliuaiminii* belongs to Astragalussect.Laguropsis (A.subg.Calycocystis) by its acaulescent, densely caespitose, with only white hairs. The calyx expands to become saccate, enveloping the pod.

**Figure 1.**
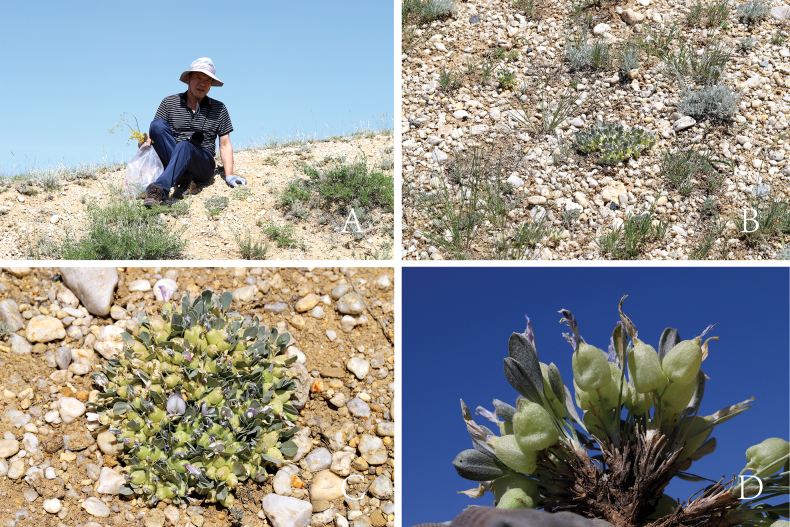
Plants and habitat of *Astragalusliuaiminii* Z. Z. Yang and Q. R. Liu **A** Mr. Aimin Liu and the distribution area **B** habitat **C** plant **D** side view of the plant.

#### Type.

China. XinJiang Province. Habahe County. Mt. Talede, 1050 m elev., 9 June 2023, on dry gravelly slopes, *A. M. Liu, Z. Z. Yang 2388* (Holotype BNU!)

**Figure 2.**
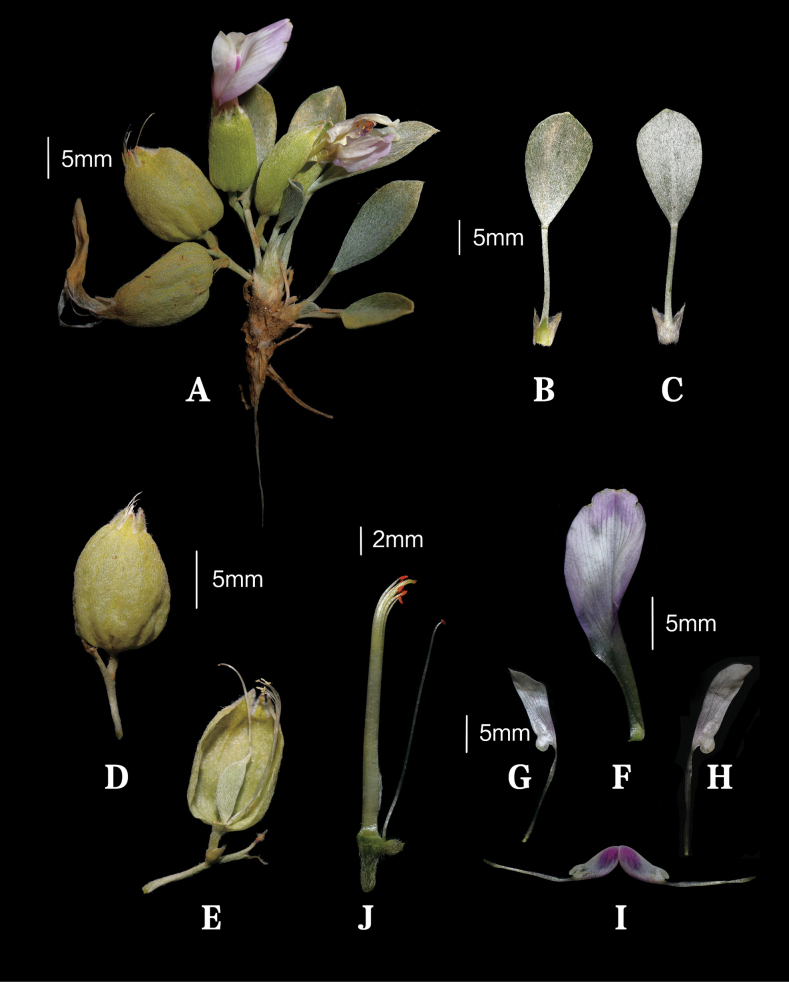
Habit and plant structures of *Astragalusliuaiminii* Z. Z. Yang & Q. R. Liu **A** habit **B** leaf, adaxial view **C** leaf, abaxial view **D** mature saccate calyx **E** half calyx removed exposing immature fruit **G–H** wings **I** keel **J** pistils and stamens.

#### Paratypes.

China. Xinjiang: Habahe County, 1000 m elev., 11 June 2023, *A. M. Liu, Z. Z. Yang 2398* (BNU!).

#### Description.

Plants perennial, 3–5 cm tall, acaulescent or nearly so, densely caespitose, covered with white medifixed hairs. Caudex with a pluricipital root crown. Stipules whitish membranous, 3–5 mm long, triangular, adnate to the petiole for ca. 2 mm, densely covered with appressed white hairs. Leaves with single leaflet, 2–3.5 cm long, rhomboid, petiole 0.8–1.5 cm long, very densely covered with appressed white hairs; leaflets obovate to elliptic, subacute at apex, rather densely covered with appressed hairs on both surfaces. Racemes 1–2 flower, 1.2–2.2 cm. Bracts whitish membranous, narrowly triangular, ca. 2 mm long, anther densely covered with short appressed hairs. Pedicels very short, with white hairs. Calyx tubular at anthesis, ca. 9 mm, later enlarged and becoming saccate, spherical-ovoid, 15–17 mm, densely covered with white appressed hairs, and denser towards the margins of teeth; teeth triangular, 2–3 mm long. Petals whitish or pinkish. Standard 25–27 mm long; limb oblong, 6.5–8 mm wide, emarginate, constricted below the middle, gradually narrowing into the claw. Wings 23–24 mm long; limbs narrowly oblong, obtuse, ca. 10–11 × 1.2 mm; claw ca. 13 mm long. Keel ca. 19–20 mm long; limbs narrowly elliptic, with widely-curved lower edge and straight upper edge, pink-purple at apex, ca. 13–14 × 2.2 mm; claw ca. 6 mm long. Ovary sessile, white hairy; style glabrous. Mature legumes not seen.

**Figure 3.**
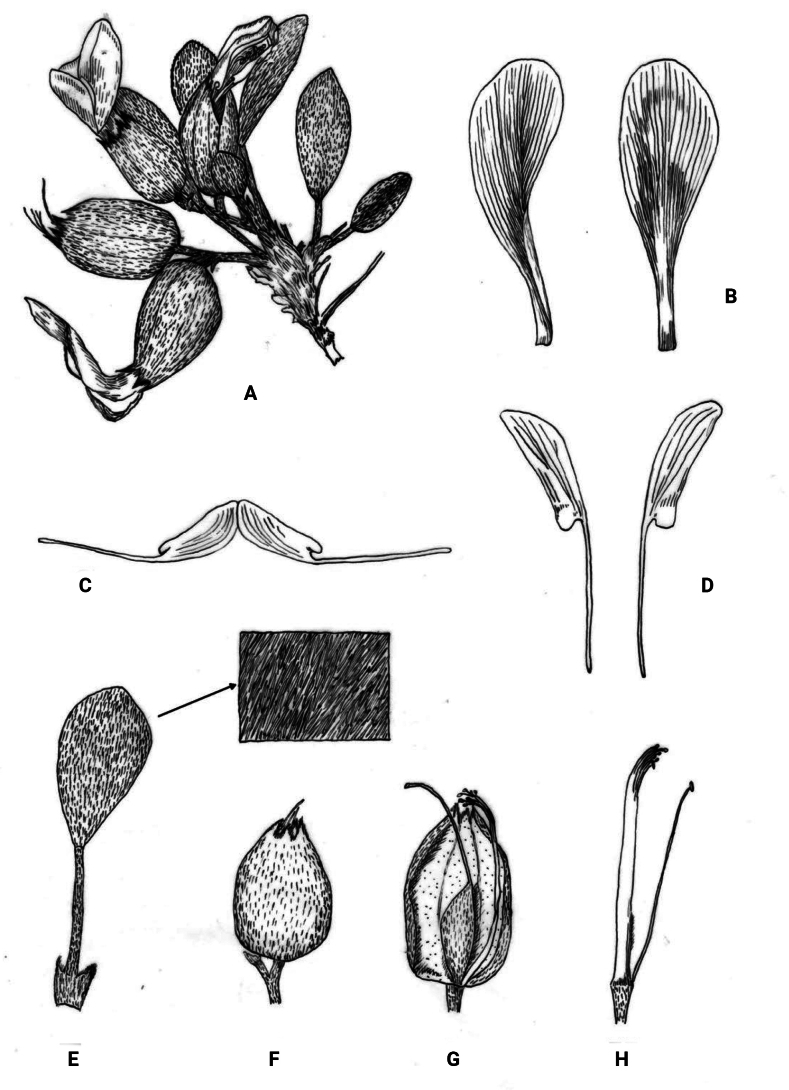
*Astragalusliuaiminii* sp. nov. **A** plants **B** standard **C** keel **D** wings **E** leaf, adaxial view **F** mature saccate calyx **G** half calyx removed exposing immature fruit **H** pistils and stamens.

#### Phenology.

Flowering in June.

#### Distribution and habitat.

*Astragalusliuaiminii* is currently known only from Habahe County in northwest Xinjiang Province, China, where it grows at an altitude of 1000–1100 m, on dry gravel slopes.

#### Etymology.

The species is named in honour of Mr. Aimin Liu, who collected the type specimens.

#### Chinese name.

爱民黄芪 (Ai Min Huang Qi).

#### Preliminary conservation status.

*Astragalusliuaiminii* has a restricted distribution area. It is only known from the upstream region of Habahe County, where there is no natural protection area. The total population size of the species is estimated no more than 100 individuals. According to the IUCN (2022), this new species should be considered as “Critically Endangered” (CR).

#### Results.

The new species is close to *A.wenquanensis* S. B. Ho, but differs chiefly in leaves having single leaflet (vs. 3–5 leaflets) and inflorescences with 1–2 flowers (vs. 5–7 flowers). It is also similar to *A.monophyllus* Maxim in leaf shape, but differs in its calyx expanding into a sac-like and totally enveloping the pod (vs. calyx tubular, and ruptured by pod as it matures). *Astragalusmonophyllus* is distributed in the northeastern and southern regions of Xinjiang, and belongs to subg. Cercidothrix, while *A.liuaiminii* is only found in Habahe County of the Altai Mountains in the northern part of Xinjiang, belonging to subg. Calycocystis. The calyx is an important taxonomic characteristic of the genus *Astragalus*, these two species have distinctly different calyces representative of different subgenera. The differences between *A.liuaiminii*, *A.wenquanensis* and *A.monophyllus* are summarised in Table 1.

**Table 1.** Morphological comparisons among *Astragalusliuaiminii*, *A.wenquanensis* and *A.monophyllus*.

Characters	*A.liuaiminii*	*A.wenquanensis*	*A.monophyllus*
Subgenera	subg. Calycocystis	subg. Calycocystis	subg. Cercidothrix
leaf	Single leaflet	3–5 leaflets	1–3 leaflets
inflorescence	1–2 flowers	5–7 flowers	1–2 flowers
calyx	light green suffused, covered with white hairs, expanding to become saccate and enveloping the pod after flowering	red suffused, covered with white and black hairs, expanding to become saccate and enveloping the pod after flowering	calyx tubular, not expanding and becoming saccate, and ruptured by pod after flowering

## Supplementary Material

XML Treatment for Astragalus liuaiminii

## ﻿Additional information

### Author ORCIDs

Zong-Zong Yang https://orcid.org/0000-0003-3618-6970

### Data availability

All of the data that support the findings of this study are available in the main text.
